# Complete Genome and Plasmid Sequences of the Psychrotolerant *Aureimonas* Strain SA4125, Isolated from Antarctic Moss Vegetation

**DOI:** 10.1128/MRA.00878-21

**Published:** 2021-10-14

**Authors:** Hiroyuki D. Sakai, Ryo Matsuda, Satoshi Imura, Norio Kurosawa

**Affiliations:** a Department of Science and Engineering for Sustainable Innovation, Faculty of Science and Engineering, Soka University, Hachioji, Tokyo, Japan; b Department of Environmental Engineering for Symbiosis, Graduate School of Science and Engineering, Soka University, Hachioji, Tokyo, Japan; c National Institute of Polar Research, Research Organization of Information and Systems, Tokyo, Japan; d Department of Polar Science, School of Multidisciplinary Sciences, The Graduate University for Advanced Studies, SOKENDAI, Tokyo, Japan; University of Arizona

## Abstract

The complete genome sequences of *Aureimonas* sp. strain SA4125 and its native plasmid pSA4125 were determined. The genome sequence comprises 4,968,066 bp, with a GC content of 66.0%, and contains 4,691 coding DNA sequences (CDSs), 3 rRNA operons, and 50 tRNAs. The native plasmid comprises 131,777 bp, with a GC content of 62.3%, and contains 138 CDSs.

## ANNOUNCEMENT

Members of the genus *Aureimonas* are aerobic, Gram stain-negative, rod-shaped, motile, and aerobic bacteria having Q-10 as the dominant isoprenoid quinone (1). They have been isolated from a variety of environments, such as caves (2), air samples (3), water-cooling systems (4), rusty iron plates (5), the leaves or bark of plants (6–10), a melt pond on sea ice (11), and an ice core (1). We isolated strain SA4125, belonging to the genus *Aureimonas*, from Antarctic moss vegetation. Here, we report the complete genome sequences of SA4125 and its native plasmid, pSA4125.

Strain SA4125 was isolated from soil associated with moss vegetation collected at Langhovde ice-free area in Antarctica. The soil sample was suspended in saline, serially diluted, and spread onto 0.7% Gelrite plates containing modified Brock’s basal salt (MBS) (12) and 0.1% yeast extract (pH 7.6). After 2 weeks of incubation at 4°C, a small yellow colony was picked up and purified by repeated single-colony isolation.

For DNA extraction, SA4125 was cultivated in MBS and 0.1% yeast extract (pH 8.0) at 20°C; 500 ml of log-phase culture was centrifuged (8,000 × *g*, 4°C, 15 min). Genomic DNA was extracted from the cell pellet using Genomic-tips 100/G (Qiagen). This DNA was used for both short- and long-read sequencing. For short-read sequencing, a DNA library was constructed using the NEBNext Ultra II FS DNA library prep kit for Illumina (New England BioLabs) and sent to Novogene Bioinformatics Technology (China) to be sequenced on an Illumina NovaSeq 6000 platform (2 × 150 bp). The short reads were quality filtered using fastp v.0.20.1 (13). For long-read sequencing, a DNA library was prepared following the protocol as described by Oxford Nanopore Technologies (ONT) (NBE_9065_v109_revZ_14Aug2019) and applied to a MinION sequencer with a R9 flow cell. Base calling was conducted using Guppy v.4.3.4, implemented in MinKNOW v.4.2.8 software (ONT). The long reads were quality filtered using Filtlong v.0.2.0 (https://github.com/rrwick/Filtlong). Hybrid assembly was conducted using Unicycler v.0.4.8 (14) with all the quality-filtered reads. The obtained genome and plasmid sequences were annotated using DFAST v.1.4.0 (15). The genome was rotated using Unicycler v.0.4.8 (14) so that the *dnaA* gene came first. Default parameters were used for all software.

The sequencing metrics are summarized in Table 1. As a result of hybrid assembly, we obtained one circular chromosome (4,968,066 bp; GC content, 66.0%) and one plasmid, named pSA4125 (131,777 bp; GC content, 62.3%). The chromosome contained 4,691 coding DNA sequences (CDSs), 3 rRNA operons, and 50 tRNAs. Based on the average nucleotide identity (ANI) calculated using DFAST v.1.4.0 (15), the closest species to SA4125 was Aureimonas glaciei (1), with an ANI value of 86.9%, suggesting that strain SA4125 may be a novel species. Genes for a cold shock protein (5 copies of *cspA*) (16), trehalose synthesis (*otsB*, *treS*), and DEAD box helicase (17) were found, suggesting that SA4125 is a cold-adapted bacteria. Plasmid pSA4125 has 138 CDSs, containing genes for transposase (13 copies), conjugal transfer proteins (*traBDGHIR*, *trbBCDEFGHIJL*), and a replication initiation protein (*repC*). The sequence data presented here will contribute to taxonomic and comparative genomic studies of the genus *Aureimonas*.

**TABLE 1:** Sequencing metrics

Characteristic	Data for:	
	NovaSeq 6000	MinION
Before quality filtering		
No. of raw reads	8,806,588	87,131
Total size (bp)	1,320,988,200	1,254,271,517
*N*_50_ (bp)	150	25,594
After quality filtering^*a*^		
No. of reads	8,782,920	87,553
Total size (bp)	1,203,055,702	1,000,007,876
*N*_50_ (bp)	150	17,382

a The commands used for the quality filtering are as follows. For fastp, “fastp -i read1.fastq -I read2.fastq -o filtered_read1.fastq -O filtered_read2.fastq.” For Filtlong, “filtlong -1 filtered_read1.fastq -2 filtered_read2.fastq --min_length 1000 --target_bases 1000000000 --trim --split 100 --mean_q_weight 10 long_read.fastq > filtered_long_read.fastq.”

### Data availability.

The genome sequences of strain SA4125 and plasmid pSA4125 have been deposited in DDBJ/ENA/GenBank under the accession numbers AP025032 and AP025033, respectively. The raw reads have been deposited in the SRA under the accession numbers DRR287488 and DRR287489.
